# Uterine Preservation and Maternal Outcomes Across FIGO Severity in Placenta Accreta Spectrum: A Six-Year Retrospective Cohort in Vietnam

**DOI:** 10.3390/medsci14060683

**Published:** 2026-10-19

**Authors:** Trang Nguyen-Khanh Huynh, Tuyet Thi-Diem Hoang, Dung Tuan Dao, Ha Thanh Ngo, Linh Dieu Pham, Minh Phuc-Khanh Huynh, Huy Phuong Tran

**Affiliations:** 1 Hung Vuong Hospital, Ho Chi Minh City 700000, Vietnam; 2 Department of Obstetrics and Gynecology, Pham Ngoc Thach University of Medicine, Ho Chi Minh City 700000, Vietnam

**Keywords:** placenta accreta spectrum, uterine preservation, hysterectomy, blood loss, surgical, blood transfusion, cesarean section

## Abstract

**Background/Objectives:** Uterus-preserving management of placenta accreta spectrum (PAS) is increasingly reported, but preservation rates are difficult to interpret without considering disease severity and maternal morbidity. We aimed to estimate short-term uterine preservation through hospital discharge and describe maternal outcomes by FIGO clinical severity. **Methods:** We conducted a retrospective single-center cohort study of 291 women with clinically diagnosed PAS and intraoperative FIGO grading who underwent cesarean delivery at Hung Vuong Hospital, a tertiary maternity referral hospital in Vietnam, from 2019 to 2024. The primary outcome was short-term uterine preservation through hospital discharge, defined as no hysterectomy during the index operation or before discharge. Secondary and exploratory analyses described hemorrhage and maternal morbidity across FIGO severity. **Results:** Short-term uterine preservation through hospital discharge was achieved in 282/291 women (96.9%; 95% CI 94.2–98.4), including 72/79 with FIGO grade 3 (91.1%; 95% CI 82.8–95.6). Major hemorrhage occurred in 81/291 (27.8%) and severe maternal morbidity in 25/291 (8.6%); no maternal deaths occurred. In FIGO grade 3, major hemorrhage occurred in 51/79 (64.6%), high-volume red-cell transfusion in 37/79 (46.8%), and severe maternal morbidity in 21/79 (26.6%). **Conclusions:** Most women retained the uterus through hospital discharge in this cohort, but women with FIGO grade 3 had high rates of hemorrhage, transfusion, and maternal morbidity. Without a concurrent planned-hysterectomy comparator, this study cannot compare the effectiveness of uterine preservation with hysterectomy.

## 1. Introduction

Placenta accreta spectrum (PAS) is a high-risk obstetric condition associated with severe hemorrhage, transfusion, organ injury, hysterectomy, and maternal morbidity. Current guidance recommends antenatal diagnosis, delivery in experienced centers, multidisciplinary care, and ready access to blood products. Cesarean hysterectomy with the placenta left in situ remains the standard approach for many women with significant PAS [1,2,3]. The International Federation of Gynecology and Obstetrics (FIGO) clinical classification provides a standardized way to describe PAS severity at surgery when complete histopathology is not available [4].

Previous cesarean delivery may disrupt the endometrial–myometrial interface and allow implantation within the cesarean scar. Cesarean scar pregnancy has been linked to PAS when the pregnancy continues beyond early gestation [5,6].

PAS encompasses a clinically heterogeneous group of disorders, ranging from abnormally adherent placenta to deeply invasive disease involving the uterine serosa or adjacent organs. This heterogeneity matters because the technical difficulty of surgery, anticipated blood loss, transfusion exposure, and risk of organ injury can differ across the spectrum. The FIGO clinical classification was developed to provide a common intraoperative framework when complete histopathologic assessment is unavailable, which is particularly relevant when uterine preservation limits the amount of tissue available for pathologic verification [1,4]. Consequently, outcome reporting that combines all PAS cases without a measure of clinical severity may obscure important differences in procedural risk and resource requirements.

Uterus-preserving management may include leaving the placenta in situ, focal or myometrial resection with reconstruction, vascular-control procedures, and other hemostatic techniques. Although favorable outcomes have been reported in selected patients, studies vary in patient selection, technique, disease severity, operator experience, and outcome definitions [7,8,9,10,11,12]. Preservation rates are therefore difficult to interpret without considering disease severity and the criteria for rescue hysterectomy.

Uterine preservation is not a single operation. Depending on anatomy, bleeding, local expertise, and patient priorities, management may combine placental-bed hemostasis, myometrial resection or reconstruction, vascular-control procedures, compression sutures, tamponade, or delayed management [7,8,9,10,11,12]. Direct comparison across studies is difficult because treatment selection depends on PAS severity and center expertise. Preservation rates should therefore be interpreted alongside hemorrhage, transfusion, organ injury, maternal morbidity, and rescue hysterectomy.

These questions are relevant in middle-income settings, where recommended multidisciplinary resources may not be uniformly available [13,14]. Conservative PAS surgery has also been reported in Vietnam [15]. We therefore evaluated short-term uterine preservation through hospital discharge and maternal outcomes by FIGO severity at a tertiary referral hospital in Vietnam. The study was descriptive and exploratory; FIGO severity was used to describe clinical context rather than as a causal exposure.

## 2. Materials and Methods

### 2.1. Study Design and Participants

We conducted a retrospective single-center cohort study at Hung Vuong Hospital, a tertiary maternity referral hospital in Ho Chi Minh City, Vietnam, from January 2019 to December 2024. The cohort included all 291 eligible women, with each woman contributing once at the index delivery admission. PAS was assessed antenatally using clinical risk factors and imaging. At surgery, the experienced operating surgeon diagnosed PAS and assigned the FIGO grade based on intraoperative findings. According to the FIGO clinical classification, grade 1 indicated abnormal placental adherence without visible invasion; grade 2 was characterized by abnormal placental-bed appearance, including uterine bulging or hypervascularity, without placental tissue extending through the uterine serosa; grade 3A indicated placental tissue extending through the uterine serosa without bladder involvement; grade 3B indicated bladder invasion; and grade 3C indicated invasion of other pelvic tissues or organs [4]. The research team extracted the findings and FIGO grade from the records. Histopathology was supportive when tissue was available but was not required because uterus-preserving procedures often did not provide full-thickness placental-bed tissue. Some grade 1 cases may have been misclassified when histopathology was unavailable. No formal power calculation was performed because all eligible women during the study period were included. Reporting followed STROBE [16].

### 2.2. PAS Management

The institutional PAS management framework included both uterine preservation and hysterectomy. Antenatal care included risk assessment, Doppler ultrasound, selective magnetic resonance imaging (MRI), counseling, blood-bank and anesthetic preparation, neonatal planning, and surgical consultation when needed. At surgery, management was guided by PAS severity, bleeding, maternal condition, and the feasibility of uterine preservation. Hysterectomy was performed for uncontrolled bleeding, hemodynamic instability, coagulopathy, or when uterine preservation was not feasible. The institutional protocol was used throughout the study period.

Management was individualized, and more than one hemostatic or reconstructive technique could be used in the same operation. These components therefore overlapped and were not analyzed as separate interventions. Uterine preservation was attempted in all 291 women, with hysterectomy performed when preservation was unsuccessful or unsafe. Details of the recorded operative components are provided in Supplementary Table S1.

### 2.3. Outcomes

The primary outcome was short-term uterine preservation through hospital discharge, defined as no hysterectomy during the index operation or before discharge. Women who retained the uterus at surgery but underwent hysterectomy later during the same admission were counted as preservation failures. Preservation at the end of the index operation was reported separately (Supplementary Table S2).

Maternal outcomes included estimated blood loss (EBL), major hemorrhage, red-cell transfusion, high-volume red-cell transfusion, documented massive transfusion, operative duration, bladder injury requiring repair, postoperative infection, unplanned non-hysterectomy reintervention, postoperative length of stay, severe maternal morbidity (SMM), and maternal death. Major hemorrhage was defined as intraoperative EBL ≥ 2000 mL. High-volume red-cell transfusion was defined for descriptive purposes as a cumulative packed red-cell volume ≥ 1500 mL during the index admission. Documented massive transfusion was identified separately from operative, anesthesia, or transfusion records. Red-cell transfusion was recorded throughout the index admission. Transfusion decisions were individualized according to blood loss, hemodynamic status, hemoglobin status, anticipated bleeding risk, and clinical judgment; preoperative anemia could also be considered when delivery was imminent. EBL was obtained retrospectively from operative and anesthesia records and reflected the clinical estimate based on suction-canister volume, surgical sponges, and visual assessment of the operative field. A standardized quantitative blood-loss protocol was not used consistently throughout the study period. The study-specific SMM composite included maternal death, documented massive transfusion, cardiopulmonary arrest requiring resuscitation, bladder injury requiring surgical repair, or unplanned non-hysterectomy reintervention before discharge. Hysterectomy was excluded from the composite because it was part of the primary outcome. This study-specific composite differs from the 2025 PAS-specific standardized SMM criteria [17].

Gestational age at delivery and delivery urgency were obtained from clinical records. Preoperative detection of PAS was based on the overall clinical assessment using available antenatal information, including ultrasound, MRI when performed, referral information, and documented risk factors. Emergency delivery could reflect bleeding, labor, fetal concerns, or other urgent indications, so its associations with outcomes were considered exploratory.

### 2.4. Hospital Charges

Hospital charges were analyzed as a supplementary outcome. Charges were standardized to 2024 Vietnamese dong and reflect hospital billing rather than economic costs or cost-effectiveness.

### 2.5. Statistical Analysis

Categorical variables were summarized as n/N (%) and continuous variables as median (interquartile range), where appropriate. The primary outcome was reported as the proportion of women with short-term uterine preservation through hospital discharge with two-sided Wilson 95% confidence intervals (CIs), overall and by FIGO grades 1, 2, and 3. Wilson intervals were used because preservation failures were uncommon. Baseline characteristics were descriptive, and no *p* values were calculated for comparisons across FIGO severity groups.

Exploratory regression analyses were limited to a small number of relevant predictors because of the number of outcome events. FIGO grades 3A–3C were combined as grade 3, and grade 3 was compared with grades 1 and 2 combined. Emergency delivery was compared with scheduled delivery, and placenta previa/low-lying placenta with its absence. These models were intended to describe associations rather than estimate causal effects or develop a prediction model.

Preservation failure was analyzed using Firth penalized logistic regression with FIGO grade 3 and emergency delivery. Because only nine preservation failures occurred, these estimates were interpreted cautiously. SMM was analyzed using Firth regression with FIGO grade 3, emergency delivery, and placenta previa/low-lying placenta [18]. Firth regression was used for these sparse outcomes to reduce instability from complete or near-complete separation, with profile penalized-likelihood 95% CIs. Major hemorrhage was analyzed using standard logistic regression with the same three predictors. Median EBL was analyzed using quantile regression at τ = 0.50 with the same predictors because EBL was right-skewed [19].

Sensitivity analyses examined short-term uterine preservation after excluding FIGO grade 1, within FIGO grade 3, among women with histopathology recorded, and among women whose histopathology was compatible with PAS. A post hoc temporal sensitivity analysis compared 2019–2021 with 2022–2024 for uterine preservation through discharge, hysterectomy through discharge, EBL, and major hemorrhage; FIGO grade 3 was also summarized to describe case mix. Missing data were handled by complete-case analysis for the relevant outcome or model, with available denominators reported when they differed from the full cohort. Analyses were performed in R version 4.6.1.

### 2.6. Ethics

The study was approved by the Hung Vuong Hospital Committee (Decision No. 4074/QĐ-BVHV, dated 24 June 2025; study code CS/HV/25/32), which waived the requirement for informed consent.

## 3. Results

### 3.1. Study Population

**Figure 1:**
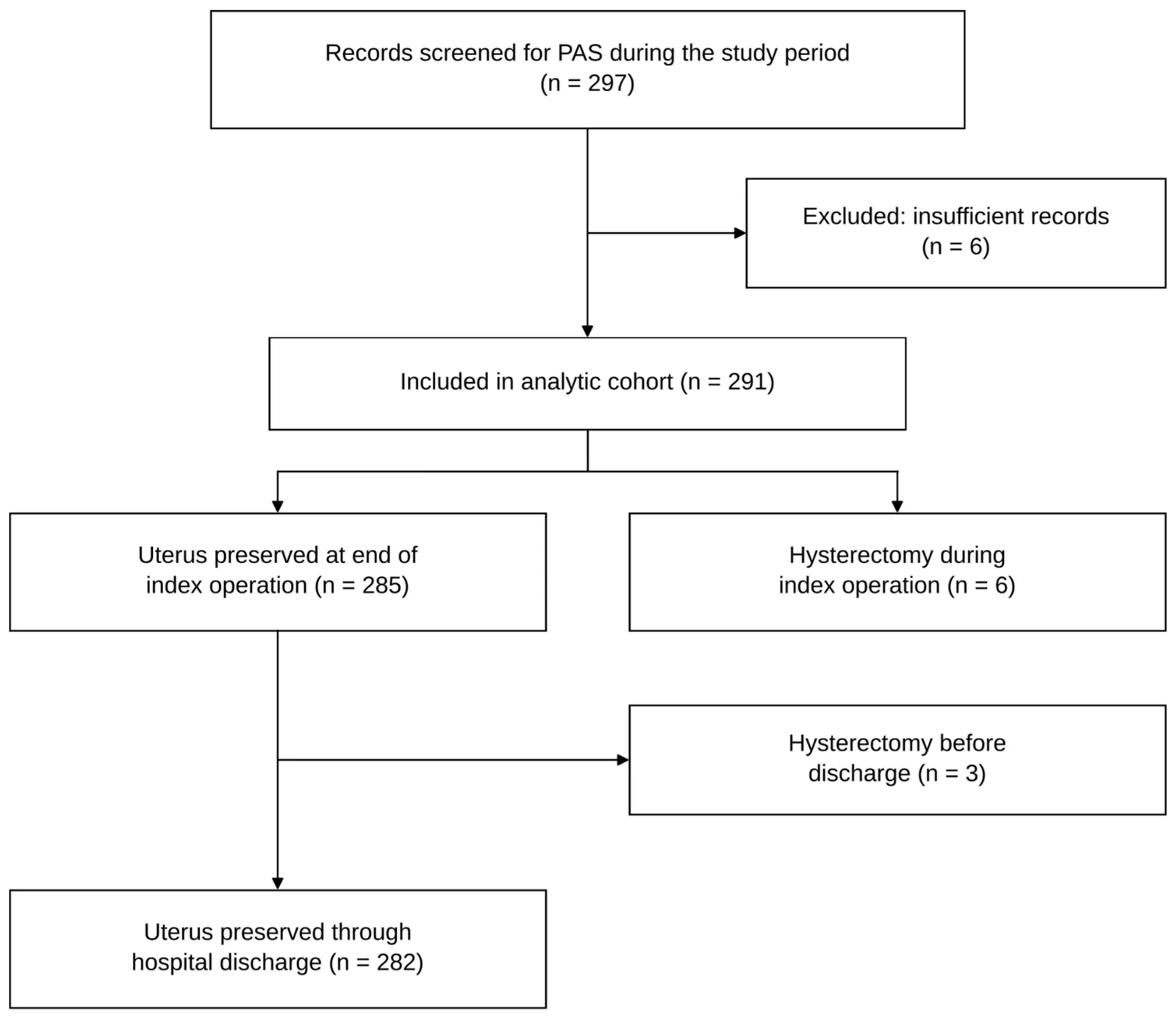
Study flow diagram. PAS = placenta accreta spectrum.

A total of 297 records were screened; 6 were excluded because of insufficient records, leaving 291 eligible women (Figure 1). The cohort included 88 women with FIGO grade 1, 124 with grade 2, and 79 with grade 3 PAS. Baseline characteristics are shown in Table 1, with diagnostic, operative, and data-availability details in Supplementary Table S1.

Antenatal and operative details are summarized in Supplementary Table S1. MRI was performed in 215/291 women (73.9%), and the clinical dataset recorded multiple ultrasound features associated with PAS. Operative management involved overlapping uterus-preserving components: placental-bed hemostatic suturing was recorded in 277/291 women (95.2%), any arterial ligation in 274/291 (94.2%), and focal myometrial area-reduction or suturing in 171/291 (58.8%). These frequencies describe the care delivered and should not be interpreted as comparative effectiveness estimates for individual techniques. Primary analytical variables, including final uterine status, FIGO grade, EBL, transfusion variables, and SMM, were complete for all 291 women; missingness was limited for in-hospital secondary outcomes but was greater for post-discharge observations (Supplementary Table S1).

Median maternal age was 35.0 years (IQR 31.0–38.0), and 227/291 women (78.0%) had a history of uterine surgery. Placenta previa or a low-lying placenta was present in 239/291 (82.1%), and PAS was identified preoperatively in 262/291 (90.0%). Prior uterine surgery was more common in FIGO grade 3 (93.7%) than in grades 1 and 2 (70.5% and 73.4%), whereas emergency delivery was similar across FIGO strata (Table 1). These characteristics are descriptive and were not used to define the severity groups.

### 3.2. Primary Outcome and Maternal Morbidity

**Figure 2:**
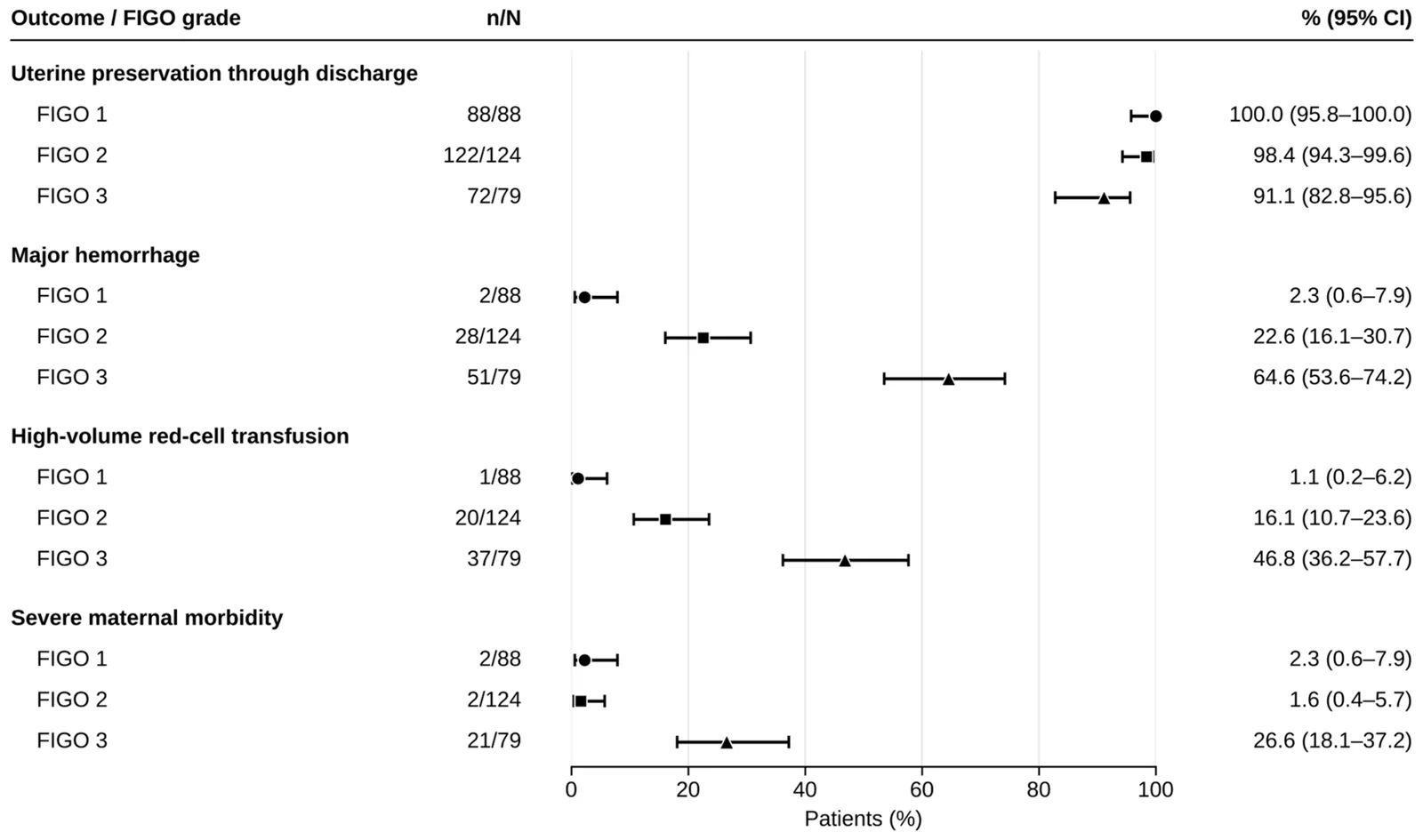
Clinical outcomes by FIGO severity. Points show percentages and error bars show Wilson 95% confidence intervals for short-term uterine preservation through hospital discharge, major hemorrhage, high-volume red-cell transfusion, and severe maternal morbidity across FIGO grades 1–3. FIGO grade 3 includes grades 3A–3C. FIGO = International Federation of Gynecology and Obstetrics; CI = confidence interval; and n/N = number of events/number assessed.

Short-term uterine preservation through hospital discharge was achieved in 282/291 women (96.9%; 95% CI 94.2–98.4): 88/88 (100.0%) in FIGO grade 1, 122/124 (98.4%) in grade 2, and 72/79 (91.1%; 95% CI 82.8–95.6) in grade 3 (Table 2, Figure 2). Median EBL increased from 600 mL (400–900) in grade 1 to 2500 mL (1550–3500) in grade 3. Major hemorrhage occurred in 81/291 women (27.8%), including 51/79 (64.6%) with grade 3. High-volume red-cell transfusion occurred in 58/291 (19.9%) and 37/79 (46.8%) with grade 3. SMM occurred in 25/291 (8.6%), including 21/79 (26.6%) with grade 3; all eight bladder injuries occurred in grade 3. No maternal deaths occurred. Additional maternal outcomes are reported in Supplementary Table S2.

Preservation at completion of the index operation was 285/291 (97.9%) (Supplementary Table S2). All six intraoperative hysterectomies followed unsuccessful preservation attempts, due to firmly adherent placenta (*n* = 4) or bleeding control (*n* = 2). Three additional women underwent hysterectomy within 24 h of cesarean delivery: two for postpartum hemorrhage unresponsive to treatment and one for internal bleeding associated with a hematoma adjacent to the uterine incision. The overall median RBC transfusion volume was 500 mL (IQR 250–1400) and increased to 1400 mL (700–2500) in FIGO grade 3. Operative duration also rose with severity, from a median of 58 min (48–70) in grade 1 to 95 min (78–121) in grade 3. Postoperative infection was documented in 23/290 women (7.9%), and five women (1.7%) required an unplanned non-hysterectomy reintervention. Median postoperative length of stay was 5 days (IQR 4–6) overall and 6 days (IQR 5–7.8) in grade 3.

### 3.3. Delivery Urgency and Gestational Age

Delivery was scheduled in 200 women and an emergency in 91. Median gestational age was lower with emergency than scheduled delivery (35.3 weeks (IQR 33.0–36.7) vs. 37.0 weeks (36.9–37.3)); this difference is descriptive and not interpreted causally (Supplementary Table S3).

### 3.4. Robustness and Exploratory Models

Short-term uterine preservation was 194/203 (95.6%; 95% CI 91.8–97.7) after excluding FIGO grade 1 and 72/79 (91.1%; 95% CI 82.8–95.6) in FIGO grade 3. In the histopathology-recorded subset, preservation was 79/88 (89.8%; 95% CI 81.7–94.5), and among women with histopathology compatible with PAS, it was 74/83 (89.2%; 95% CI 80.7–94.2) (Supplementary Table S4). Five of the 88 histopathology results were non-diagnostic due to sampling problems (FIGO grade 1, *n* = 1; grade 2, *n* = 2; grade 3, *n* = 2), rather than documented as negative for PAS. Because histopathology was available selectively, these analyses were considered descriptive sensitivity analyses.

In exploratory adjusted models, FIGO grade 3 was associated with higher odds of preservation failure (OR 9.65; 95% CI 2.46–53.48), SMM (OR 17.63; 95% CI 6.61–58.26), and major hemorrhage (OR 11.04; 95% CI 6.02–20.24), and with 1600 mL higher median EBL (95% CI 1345–1855), compared with FIGO grades 1 and 2 combined (Supplementary Table S5). Because only nine preservation failures occurred, the corresponding odds ratio was imprecise and should be interpreted as exploratory rather than as a stable independent predictor. Median total hospital charges increased from 20.96 million 2024 VND in FIGO grade 1 to 35.64 million VND in grade 3 (Supplementary Table S6). These values represent hospital billing and were not interpreted as costs or cost-effectiveness.

In the post hoc temporal analysis, short-term uterine preservation was 95.9% in 2019–2021 and 97.6% in 2022–2024; hysterectomy through discharge was 4.1% and 2.4%; median EBL was 1200 (700–2300) mL and 950 (525–1975) mL; and major hemorrhage occurred in 31.4% and 25.3%, respectively (Supplementary Table S7).

### 3.5. Post-Discharge Ascertainment

Post-discharge data availability is summarized in Supplementary Table S8. Because ascertainment was incomplete, long-term outcome rates were not estimated.

The completeness of post-discharge ascertainment varied by outcome. A scheduled follow-up visit was documented for 243/291 women (83.5%), 30-day readmission status for 193/291 (66.3%), and post-discharge hysterectomy status for 172/291 (59.1%). Reproductive outcomes were much less completely captured: spontaneous pregnancy status was available for 26/291 women (8.9%), resumption of menses for 21/291 (7.2%), and contraception status for 33/291 (11.3%). For this reason, post-discharge observations were not converted into cohort rates or used to infer long-term reproductive success.

## 4. Discussion

In this six-year cohort, most women with PAS retained the uterus through hospital discharge, including more than 90% of those with FIGO grade 3. This high preservation rate should be interpreted together with maternal morbidity. Major hemorrhage occurred in more than one quarter of the cohort, almost one fifth received high-volume red-cell transfusion, and maternal morbidity increased markedly with FIGO severity. Among women with grade 3 disease, nearly two thirds had EBL ≥ 2000 mL, almost half received high-volume red-cell transfusion, more than one quarter experienced SMM, and all bladder injuries occurred in this group. These findings show that short-term uterine preservation and maternal surgical risk describe different aspects of outcome. Retaining the uterus at discharge does not necessarily indicate an uncomplicated operation or low maternal morbidity.

The red-cell transfusion rate in FIGO grade 1 was high (50/88, 56.8%) despite a median intraoperative EBL of 600 mL. Transfusion was recorded across the admission and could reflect hemoglobin status or anticipated bleeding risk in addition to surgical blood loss. Because patient-level indications and pretransfusion hemoglobin values were not systematically collected, this finding warrants caution when comparing transfusion rates across centers.

This distinction is important when comparing our preservation rate with previous reports. Uterus-preserving management of PAS is not a single standardized procedure. PACCRETA primarily evaluated conservative management with the placenta left in situ, whereas other studies and reviews have included focal myometrial resection, uterine reconstruction, vascular-control procedures, compression techniques, or combinations of these approaches [7,8,9,10,11,12]. Patient selection also differs across studies. Some series include selected women considered suitable for preservation, whereas others include broader PAS populations with different proportions of deeply invasive disease. Definitions of successful preservation, duration of follow-up, and requirements for histopathologic confirmation also vary. A preservation rate from one cohort therefore cannot be interpreted independently of case selection, PAS severity, operative strategy, rescue criteria, and follow-up period.

Our study was not designed to compare uterine preservation with hysterectomy. Instead, it describes outcomes within an institutional framework in which both strategies were available and management was individualized according to intraoperative findings, bleeding, maternal condition, and the feasibility of preserving the uterus. This distinction is particularly important because there was no concurrent planned-hysterectomy comparison group. Women who underwent hysterectomy may have differed from those in whom preservation remained feasible. The present findings therefore describe what occurred within this clinical pathway rather than demonstrating that one management strategy is safer or more effective than another. Our approach reflects the practice of a single tertiary referral center and should not be interpreted as a recommendation to attempt uterine preservation in all patients with PAS.

Histopathologic verification requires similar caution. Preservation was lower in women with histopathology recorded and in those with histopathology compatible with PAS than in the full cohort. Histopathology was not obtained systematically, however, because uterus-preserving surgery often did not provide full-thickness placental-bed tissue. Women with available specimens may therefore have been more likely to undergo resection, hysterectomy, or other procedures that produced tissue for pathologic examination and may consequently represent a more invasive or surgically complex subgroup. At the same time, milder clinically diagnosed cases without histopathology remain more susceptible to misclassification, particularly at FIGO grade 1. The histopathology-restricted analyses should therefore not be considered an unbiased validation sample or a more accurate estimate of the cohort-wide preservation rate. Rather, the difference between these subsets illustrates the uncertainty introduced by selective pathologic verification.

The strong gradient in morbidity across FIGO severity provides an important clinical context for the preservation results. Grade 3 disease was associated with greater hemorrhage, transfusion exposure, bladder injury, operative duration, postoperative stay, and hospital charges. Urological complications are known to occur more frequently in the most invasive PAS phenotypes [20], which is consistent with all bladder injuries in our cohort occurring in grade 3. The same pattern was observed for continuous EBL and in the exploratory adjusted analyses. These findings support reporting PAS outcomes by clinical severity rather than presenting a single overall preservation rate. They should not, however, be interpreted as causal effects of FIGO grade itself, because FIGO grade is a clinical description of operative severity and anatomy rather than a modifiable exposure.

Uterine preservation also requires a clear rescue strategy. Multicenter and expert reports emphasize appropriate case selection, surgical expertise, and readiness to proceed to hysterectomy when preservation becomes unsafe or unfeasible [10,11]. In our cohort, all nine hysterectomies followed attempted uterine preservation, including three performed within 24 h for postoperative bleeding. This distinction matters because some women who retained the uterus after surgery still required hysterectomy before discharge. For this reason, we used uterine status at hospital discharge as the primary short-term outcome.

The exploratory regression analyses were consistent with the descriptive severity pattern. FIGO grade 3 was associated with preservation failure, SMM, major hemorrhage, and higher median EBL. These estimates should nevertheless be interpreted cautiously. Only nine preservation failures occurred, resulting in limited precision despite the use of Firth penalized regression. The wide confidence interval for preservation failure reflects this sparse-event setting and does not support precise estimation of the magnitude of association. The models were therefore intended to complement the severity-stratified descriptive findings rather than identify independent predictors or establish causal relationships.

Delivery urgency presents a different interpretive problem. Emergency delivery was associated with preservation failure in exploratory analysis, but emergency delivery is strongly affected by the clinical circumstances that lead to delivery. Bleeding, labor, fetal concerns, and other urgent indications may themselves be associated with more difficult surgery or worse maternal condition. A previous meta-analysis reported better maternal and neonatal outcomes after planned compared with emergency delivery in PAS [21], but such comparisons are also vulnerable to differences in underlying clinical presentation. In our cohort, emergency delivery occurred at an earlier gestational age than scheduled delivery. We therefore cannot determine whether urgency itself increased maternal risk or whether it primarily identified women who presented under less favorable clinical circumstances. The practical implication is not that emergency delivery is an independent risk factor, but that centers managing PAS should be able to provide hemorrhage control, blood products, anesthesia, experienced surgeons, and rescue hysterectomy even when a planned delivery becomes urgent.

Neonatal considerations are also relevant to delivery planning. A recent study of women with placenta previa and histopathologically confirmed PAS found greater need for neonatal resuscitation and supplemental oxygen even after adjustment for gestational age [22]. Our study was focused on maternal surgical outcomes and did not evaluate neonatal respiratory morbidity, so direct comparison is not possible. Nevertheless, the earlier gestational age observed among emergency deliveries in our cohort reinforces the importance of considering maternal and neonatal consequences together when planning delivery. Future PAS studies evaluating delivery timing should report both maternal and neonatal outcomes rather than treating them as separate clinical questions.

The setting of this study also deserves consideration. International PAS recommendations emphasize antenatal identification, referral to experienced centers, multidisciplinary planning, blood-bank support, anesthesia, and access to complex pelvic surgery [2,3]. These resources may be difficult to implement consistently in low- and middle-income settings [13]. At the same time, data from a referral center in a middle-income country have shown lower hemorrhage and transfusion after multidisciplinary PAS care was introduced [14], and conservative PAS surgery has also been reported in Vietnam [15]. Our findings add data from a high-volume Vietnamese maternity referral center, but their generalizability is therefore likely greatest for tertiary hospitals with similar surgical expertise, transfusion capacity, and access to emergency hysterectomy. The high use of blood products in our cohort further shows that uterine preservation can remain resource intensive even when hysterectomy is avoided.

Hospital charges provide another measure of this increasing resource burden. Charges were highest in FIGO grade 3, paralleling the greater blood-product use, longer operations, and longer postoperative stay in this group. These findings are descriptive and should not be interpreted as a formal economic evaluation. Hospital billing does not necessarily reflect true health-system costs, and differences in charges may be influenced by local reimbursement and accounting practices. Nevertheless, the gradient across FIGO severity reinforces the broader finding that more invasive PAS places greater demands on operative, transfusion, and inpatient resources.

Several features strengthen this study. All included women had an intraoperative FIGO clinical grade, allowing outcomes to be reported across a standardized measure of PAS severity rather than for PAS as a single heterogeneous category. The primary outcome was defined as uterine preservation through hospital discharge, and preservation at the end of the index operation was reported separately. Hemorrhage, transfusion, bladder injury, SMM, operative duration, and other maternal outcomes were also reported alongside preservation, reducing the risk of interpreting uterine retention alone as evidence of favorable maternal outcome. Sensitivity analyses excluding FIGO grade 1, restricting the analysis to grade 3, and examining histopathology-defined subsets provided additional information about the robustness and limitations of the primary estimate.

Several limitations should also be considered. First, the retrospective single-center design limits generalizability and prevents standardized prospective assessment of all clinical variables. There was no concurrent planned-hysterectomy comparator, so causal or comparative-effectiveness conclusions cannot be drawn. Second, FIGO grading depends on intraoperative clinical assessment and is therefore observer-dependent. Histopathology was available selectively, and some milder PAS cases may have been misclassified when pathologic confirmation was unavailable. Third, only nine preservation failures occurred, limiting the precision of regression analyses despite Firth penalization, and only one woman had FIGO grade 3C disease. Estimates for the most severe phenotype are therefore particularly uncertain. Fourth, our study-specific SMM composite differs from the 2025 PAS-specific standardized criteria [17], which may limit comparability with studies using those criteria.

Changes in practice over the six-year study period are another potential source of bias. Surgical experience, team organization, perioperative preparation, and transfusion practices may have evolved over time. In the post hoc comparison of 2019–2021 with 2022–2024, preservation remained high in both periods, while median EBL and major hemorrhage were numerically lower in the later period. These findings are descriptive and cannot establish a learning-curve effect, particularly because case mix also differed and FIGO grade 3 was somewhat more frequent in the later period. Nevertheless, temporal changes should be considered when interpreting outcomes from a long retrospective cohort.

Finally, our definition of uterine preservation was short term. Follow-up after hospital discharge was incomplete, particularly for reproductive outcomes, menstrual recovery, contraception, and delayed hysterectomy. The study therefore cannot determine whether women who retained the uterus at discharge subsequently maintained normal uterine function, avoided delayed complications, or achieved future pregnancy. Short-term uterine preservation should not be used as a surrogate for preserved fertility. Prospective studies with standardized long-term follow-up, reproductive outcomes, quality-of-life measures, and clearly defined eligibility and conversion criteria are needed. Future comparative studies should also account for PAS severity and confounding by indication when evaluating uterine-preserving strategies against hysterectomy or other management approaches.

## 5. Conclusions

In this study, most women retained the uterus through hospital discharge, but women with FIGO grade 3 had high rates of hemorrhage, transfusion, and maternal morbidity. These findings describe outcomes at one center and do not show that uterine preservation is superior to hysterectomy or other management strategies.

## Figures and Tables

**Table 1:** Cohort characteristics by FIGO clinical severity.

Characteristic	Overall (*n* = 291)	FIGO 1 (*n* = 88)	FIGO 2 (*n* = 124)	FIGO 3 (*n* = 79)
Maternal age, y, median [IQR]	35.0 [31.0–38.0]	34.0 [30.0–37.0]	35.0 [32.0–38.2]	36.0 [32.0–39.0]
Preoperative BMI, kg/m^2^, median [IQR]	26.3 [24.0–28.9]	26.3 [24.3–29.0]	26.1 [23.6–28.9]	26.6 [24.5–28.8]
Prior cesarean deliveries, median [IQR]	1.0 [1.0–2.0]	1.0 [0.0–1.0]	1.0 [0.0–2.0]	2.0 [1.0–2.0]
Any prior uterine surgery, n/N (%)	227/291 (78.0%)	62/88 (70.5%)	91/124 (73.4%)	74/79 (93.7%)
Placenta previa/low-lying placenta, n/N (%)	239/291 (82.1%)	71/88 (80.7%)	99/124 (79.8%)	69/79 (87.3%)
PAS detected preoperatively, n/N (%)	262/291 (90.0%)	79/88 (89.8%)	109/124 (87.9%)	74/79 (93.7%)
Gestational age at surgery, wk, median [IQR]	37.0 [36.4–37.3]	36.9 [36.4–37.1]	37.0 [36.4–37.4]	37.0 [36.2–37.2]
Emergency delivery, n/N (%)	91/291 (31.3%)	27/88 (30.7%)	41/124 (33.1%)	23/79 (29.1%)

Data are median [IQR] or n/N (%). FIGO = International Federation of Gynecology and Obstetrics; BMI = body mass index; PAS = placenta accreta spectrum; IQR = interquartile range; wk = week; y = year; and n/N = number with the characteristic/number assessed. Baseline variables are descriptive; no p values are reported.

**Table 2:** Short-term uterine preservation through hospital discharge and key maternal outcomes by FIGO clinical severity.

Outcome	Overall (*n* = 291)	FIGO 1 (*n* = 88)	FIGO 2 (*n* = 124)	FIGO 3 (*n* = 79)
Short-term uterine preservation through hospital discharge, n/N (%)	282/291 (96.9%); 95% CI 94.2–98.4%	88/88 (100.0%); 95% CI 95.8–100.0%	122/124 (98.4%); 95% CI 94.3–99.6%	72/79 (91.1%); 95% CI 82.8–95.6%
Estimated intraoperative blood loss, mL, median [IQR]	1100 [600–2000]	600 [400–900]	1150 [700–1800]	2500 [1550–3500]
Major hemorrhage (EBL ≥ 2000 mL), n/N (%)	81/291 (27.8%)	2/88 (2.3%)	28/124 (22.6%)	51/79 (64.6%)
Any red-cell transfusion, n/N (%)	226/291 (77.7%)	50/88 (56.8%)	101/124 (81.5%)	75/79 (94.9%)
High-volume RBC transfusion (≥1500 mL), n/N (%)	58/291 (19.9%)	1/88 (1.1%)	20/124 (16.1%)	37/79 (46.8%)
Documented massive transfusion, n/N (%)	15/291 (5.2%)	0/88 (0.0%)	2/124 (1.6%)	13/79 (16.5%)
Bladder injury requiring repair, n/N (%)	8/291 (2.7%)	0/88 (0.0%)	0/124 (0.0%)	8/79 (10.1%)
Severe maternal morbidity, n/N (%)	25/291 (8.6%)	2/88 (2.3%)	2/124 (1.6%)	21/79 (26.6%)
Maternal death, n/N (%)	0/291 (0.0%)	0/88 (0.0%)	0/124 (0.0%)	0/79 (0.0%)

The primary outcome is short-term uterine preservation through hospital discharge; 95% CIs are Wilson intervals. FIGO grade 3 includes grades 3A–3C. Other outcomes are descriptive. FIGO = International Federation of Gynecology and Obstetrics; CI = confidence interval; IQR = interquartile range; EBL = estimated blood loss; RBC = red blood cell; mL = milliliter; and n/N = number of events/number assessed.

## Data Availability

The data supporting the findings of this study are available from the corresponding author upon reasonable request and subject to institutional approval. The data are not publicly available because of patient privacy and ethical restrictions.

## Supplementary Materials

The following supporting information can be downloaded at: https://www.mdpi.com/article/10.3390/medsci14060683/s1, Table S1, diagnostic/operative context and data availability; Table S2, additional maternal outcomes by FIGO clinical severity; Table S3, gestational age at delivery by delivery urgency; Table S4, sensitivity and robustness analyses; Table S5, exploratory adjusted clinical models; Table S6, total hospital charges and major charge components by FIGO severity; Table S7, post hoc sensitivity analysis by study period; and Table S8, post-discharge outcome ascertainment.
