# Supplementary material for: Uterine Preservation and Maternal Outcomes Across FIGO Severity in Placenta Accreta Spectrum: A Six-Year Retrospective Cohort in Vietnam
Source: Med Sci (Basel). 2026 Oct 19;14(6):683. doi: 10.3390/medsci14060683 (PMC13706691; doi:10.3390/medsci14060683)
Supplement: Supplementary file 1 [file medsci-14-00683-s001.zip › medsci-4603123-supplementary.pdf]

Table S1. Diagnostic/operative context and data availability.

Variable	n/N (%)
**Panel A. Antenatal diagnostic work-up**	
Ultrasound suspected accreta	105/291 (36.1%)
Ultrasound suspected increta	111/291 (38.1%)
Ultrasound suspected percreta	24/291 (8.2%)
Ultrasound suspected PAS, unclassified	9/291 (3.1%)
No PAS recorded on ultrasound classification	42/291 (14.4%)
MRI performed	215/291 (73.9%)
MRI suspected PAS among MRI performed	184/215 (85.6%)
Placental lacunae recorded	182/291 (62.5%)
Loss of clear zone	238/291 (81.8%)
Subplacental/uterovesical hypervascularity	200/291 (68.7%)
Bladder-wall interruption	17/291 (5.8%)
Thin myometrium	6/291 (2.1%)
**Panel B. Selective uterus-preserving operative components**	
Placental-bed hemostatic suturing	277/291 (95.2%)
Any arterial ligation	274/291 (94.2%)
Uterine artery ligation	270/291 (92.8%)
Internal iliac/hypogastric artery ligation	12/291 (4.1%)
Myometrial area-reduction/focal suturing	171/291 (58.8%)
B-Lynch or modified B-Lynch	18/291 (6.2%)
Transverse uterine compression suturing	29/291 (10.0%)
Intrauterine balloon tamponade	13/291 (4.5%)

Ultrasound impression categories are mutually exclusive, but individual signs and operative components may overlap. PAS = placenta accreta spectrum; MRI = magnetic resonance imaging.

**Panel C. Data availability and missingness**			
**Domain / variable**	**Available (n)**	**Missing (n)**	**Missing %**
Analytical cohort / final uterine status	291	0	0.0%
Intraoperative FIGO clinical grade	291	0	0.0%
Age, BMI, gestational age at surgery	291	0	0.0%
Previous cesarean delivery / prior uterine surgery / previa-low-lying	291	0	0.0%
Intraoperative blood loss and transfusion variables	291	0	0.0%
Severe maternal morbidity	291	0	0.0%
Postoperative infection	290	1	0.3%
Postoperative length of stay	290	1	0.3%
Neonatal birthweight and immediate outcomes	291	0	0.0%
Total hospital charges	286	5	1.7%
Scheduled follow-up visit	243	48	16.5%
30-day readmission	193	98	33.7%
Post-discharge hysterectomy status	172	119	40.9%
Spontaneous pregnancy status	26	265	91.1%
Menses resumed status	21	270	92.8%
Contraception status	33	258	88.7%

FIGO = International Federation of Gynecology and Obstetrics. BMI = body mass index.

Table S2. Additional maternal outcomes by FIGO clinical severity.

Outcome	Overall	FIGO 1	FIGO 2	FIGO 3
Uterine preservation at completion of the index operation, n/N (%)	285/291 (97.9%)	88/88 (100.0%)	123/124 (99.2%)	74/79 (93.7%)
Total RBC transfusion volume, mL, median [IQR]	500 [250–1400]	250 [0–500]	500 [350–1012]	1400 [700–2500]
Operative duration, min, median [IQR]	70 [57–90]	58 [48–70]	70 [60–88]	95 [78–121]
Postoperative infection, n/N (%)	23/290 (7.9%)	3/88 (3.4%)	10/124 (8.1%)	10/78 (12.8%)
Unplanned non-hysterectomy reintervention, n/N (%)	5/291 (1.7%)	2/88 (2.3%)	0/124 (0.0%)	3/79 (3.8%)
Postoperative length of stay, d, median [IQR]	5.0 [4.0–6.0]	4.0 [4.0–5.0]	5.0 [4.0–6.0]	6.0 [5.0–7.8]

These secondary outcomes are reported for completeness and do not replace the primary or severity-stratified outcomes. FIGO = International Federation of Gynecology and Obstetrics; RBC = red blood cell; IQR = interquartile range; mL = milliliter; min = minute; and d = day.

Table S3. Gestational age at delivery by delivery urgency.

Outcome	Overall	Scheduled (n=200)	Emergency (n=91)
Gestational age at delivery, wk, median [IQR]	37.0 [36.4–37.3]	37.0 [36.9–37.3]	35.3 [33.0–36.7]

Data are descriptive. Delivery urgency was clinically determined and is not interpreted causally. wk = week; IQR = interquartile range.

Table S4. Sensitivity and robustness analyses.

Analysis	N	Result
Short-term uterine preservation through discharge, excluding FIGO grade 1	203	194/203 (95.6%; 95% CI 91.8–97.7%)
Short-term uterine preservation through discharge, FIGO grade 3	79	72/79 (91.1%; 95% CI 82.8–95.6%)
Short-term uterine preservation through discharge, histopathology-recorded subset	88	79/88 (89.8%; 95% CI 81.7–94.5%)
Short-term uterine preservation through discharge, histopathology-compatible subset	83	74/83 (89.2%; 95% CI 80.7–94.2%)

Histopathology-restricted analyses are descriptive because tissue verification was selective. FIGO = International Federation of Gynecology and Obstetrics. CI = confidence interval.

Table S5. Exploratory adjusted clinical models.

Outcome	Predictor	Adjusted estimate (95% CI)	P value
Preservation failure	FIGO grade 3	OR 9.65 (2.46–53.48)	<0.001
	Emergency delivery	OR 4.97 (1.32–22.05)	0.018
Severe maternal morbidity	FIGO grade 3	OR 17.63 (6.61–58.26)	<0.001
	Emergency delivery	OR 2.26 (0.89–5.69)	0.085
	Placenta previa/low-lying	OR 0.89 (0.28–3.30)	0.849
Major hemorrhage (EBL ≥2000 mL)	FIGO grade 3	OR 11.04 (6.02–20.24)	<0.001
	Emergency delivery	OR 1.37 (0.72–2.60)	0.339
	Placenta previa/low-lying	OR 1.59 (0.68–3.73)	0.283
Median intraoperative blood loss	FIGO grade 3	1600 mL (1345 to 1855)	<0.001
	Emergency delivery	100 mL (-145 to 345)	0.422
	Placenta previa/low-lying	200 mL (-97 to 497)	0.186

FIGO 3 was compared with FIGO 1–2; emergency with scheduled delivery; and placenta previa/low-lying placenta with its absence. Firth logistic regression was used for preservation failure and SMM, standard logistic regression for major hemorrhage, and median quantile regression for EBL. Analyses and P values are exploratory; the preservation-failure model included nine events. FIGO = International Federation of Gynecology and Obstetrics; OR = odds ratio; and CI = confidence interval.

Table S6. Total hospital charges and major charge components by FIGO severity.

Charge component	Overall (n=286)	FIGO 1 (n=87)	FIGO 2 (n=121)	FIGO 3 (n=78)
Total hospital charges	27.10 [20.21–36.46] million VND	20.96 [17.98–33.11] million VND	24.91 [20.33–30.62] million VND	35.64 [28.52–45.58] million VND
Procedure/surgical charges	4.76 [4.46–7.88] million VND	4.76 [4.46–7.34] million VND	4.76 [4.46–7.06] million VND	4.76 [4.46–8.47] million VND
Blood-product charges	2.59 [1.48–7.02] million VND	1.46 [0.40–2.55] million VND	2.60 [2.39–5.94] million VND	8.75 [2.75–13.90] million VND
Medication/supply charges	2.76 [2.15–3.57] million VND	2.38 [1.90–3.22] million VND	2.59 [2.14–3.19] million VND	3.36 [2.68–4.57] million VND
Other billable charges	12.89 [9.00–19.23] million VND	11.38 [8.19–18.79] million VND	11.94 [8.02–16.02] million VND	17.11 [11.60–22.30] million VND

Charges are median [IQR] in million 2024 VND and reflect hospital billing, not cost analysis. FIGO = International Federation of Gynecology and Obstetrics; VND = Vietnamese dong.

Table S7. Post hoc sensitivity analysis by study period.

Outcome	2019–2021	2022–2024
Cases, n	121	170
FIGO grade 3, n/N (%)	28/121 (23.1%)	51/170 (30.0%)
Short-term uterine preservation through discharge, n/N (%)	116/121 (95.9%)	166/170 (97.6%)
Hysterectomy through discharge, n/N (%)	5/121 (4.1%)	4/170 (2.4%)
Estimated intraoperative blood loss, mL, median [IQR]	1200 [700–2300]	950 [525–1975]
Major hemorrhage (EBL ≥2000 mL), n/N (%)	38/121 (31.4%)	43/170 (25.3%)

Data are descriptive and no between-period P values are reported. FIGO = International Federation of Gynecology and Obstetrics; EBL = estimated blood loss; and IQR = interquartile range.

Table S8. Post-discharge outcome ascertainment.

Post-discharge domain	Data available, n/N (%)
Scheduled follow-up visit	243/291 (83.5%)
30-day readmission	193/291 (66.3%)
Post-discharge hysterectomy status	172/291 (59.1%)
Spontaneous pregnancy status	26/291 (8.9%)
Resumption of menses	21/291 (7.2%)
Contraception status	33/291 (11.3%)

Post-discharge data came from available clinical records. Results therefore reflect documented observations only.
